# *Tropheryma whipplei* Crystalline Keratopathy: Report of a Case and Updated Review of the Literature

**DOI:** 10.1155/2012/707898

**Published:** 2012-09-04

**Authors:** Scott D. Schoenberger, Sumeer Thinda, Stephen J. Kim

**Affiliations:** Vanderbilt Eye Institute, Vanderbilt University Medical Center, Nashville, TN 37232, USA

## Abstract

*Purpose*. To report a case of *Tropheryma whipplei* infection with crystalline keratopathy and review the recent literature on the presentation, diagnosis, and management of Whipple's disease. *Methods*. Detailed case presentation and extensive literature search of Pubmed for all years through February 2012 using the following search terms: *Whipple's disease, Tropheryma whipplei, corneal deposits, crystalline keratopathy, and uveitis*. Relevant articles were retrieved and analyzed. English abstracts were used for non-English articles. Cross-referencing was employed and reference lists from selected articles were used to identify additional pertinent articles. *Results*. Diagnosis of Whipple's disease remains challenging and untreated infection can result in mortality. Ocular signs and symptoms are usually nonspecific, but several independent cases have reported the presence of intraocular crystals or crystalline-like deposits. *Conclusions*. The presence of intraocular crystals or crystalline-like deposits may be an identifying feature of ocular Whipple's disease.

## 1. Introduction

Whipple's disease is a chronic multiorgan bacterial infection due to *Tropheryma whipplei* with a variable clinical presentation and poor prognosis if untreated [1–3]. Diagnosis is challenging and the mean time from symptom onset to diagnosis is 6.4 years [2]. We report herein a patient who initially presented with vitritis and a striking crystalline keratopathy several years before the diagnosis of Whipple's disease and his untimely death. Our primary intent is to discuss the unique aspects of our case and review the available literature to determine identifiable ocular features of Whipple's disease that may aid in diagnosis of future cases. Our secondary intent is to provide an updated review of the systemic and ocular manifestations of Whipple's disease and recent advances in diagnosis and treatment.

## 2. Report of a Case

A 49-year-old male presented for evaluation of recurrent vitritis, floaters, and decreased vision of the right eye over the preceding year. Past ocular history was significant for laser assisted in situ keratomileusis (LASIK) of both eyes (OU) 7 years before. Past medical history was notable for carpal tunnel surgery 20 years ago, a right ankle fracture decades ago, and an episode of abdominal shingles several years ago. He had a questionable diagnosis of rheumatoid arthritis due to right knee swelling and distal phalangeal joint disease. He had a history of right ankle swelling and scarring and had undergone two arthroscopies of his right ankle 4–6 years prior to presentation. There was no family history of ocular or autoimmune disease. He did not smoke, drank alcohol occasionally, and did not use illicit drugs. He had extensive travel history outside the US including to the Middle East, Europe, and Asia when he was in the military.

**Figure 1:**
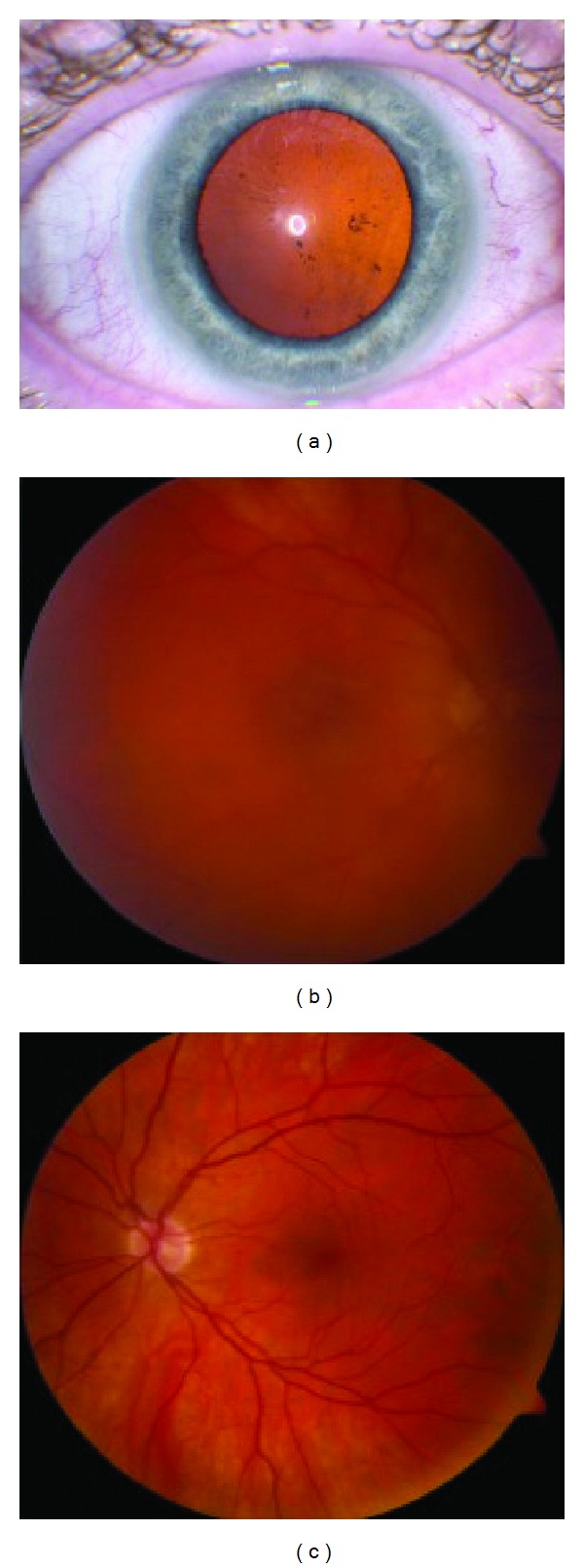
Photographs at initial presentation. (a) Slit lamp photography demonstrates deposits on the anterior lens surface of the right eye. (b) Fundus photography of the right eye shows vitreous debris without other posterior segment abnormalities. (c) Fundus photography of the left eye is unremarkable.

Examination demonstrated best corrected visual acuity of 20/50 OD and 20/20 OS. Pupillary exam and intraocular pressures were normal. Slit-lamp examination OD showed 1+ conjunctival injection, stromal scarring at the LASIK flap interface, 2+ cells in the anterior chamber, and pigment along with some white deposits on the anterior lens surface (Figure 1). His left eye exam was normal. Dilated fundus examination OD revealed vitreous debris but no retinal vasculitis or other chorioretinal lesions (Figure 1). Fluorescein angiography was unremarkable. He was diagnosed with anterior and intermediate uveitis OD and a systemic workup was initiated. Rapid plasma regain (RPR), angiotensin-converting enzyme (ACE), and HLA-B27 were negative, erythrocyte sedimentation rate (ESR) was elevated at 46, and a chest X-ray showed sequela of old granulomatous disease. Polymerase chain reaction (PCR) testing on aqueous fluid for herpes simplex virus (HSV) and varicella zoster virus (VZV) was negative. 

**Figure 2:**
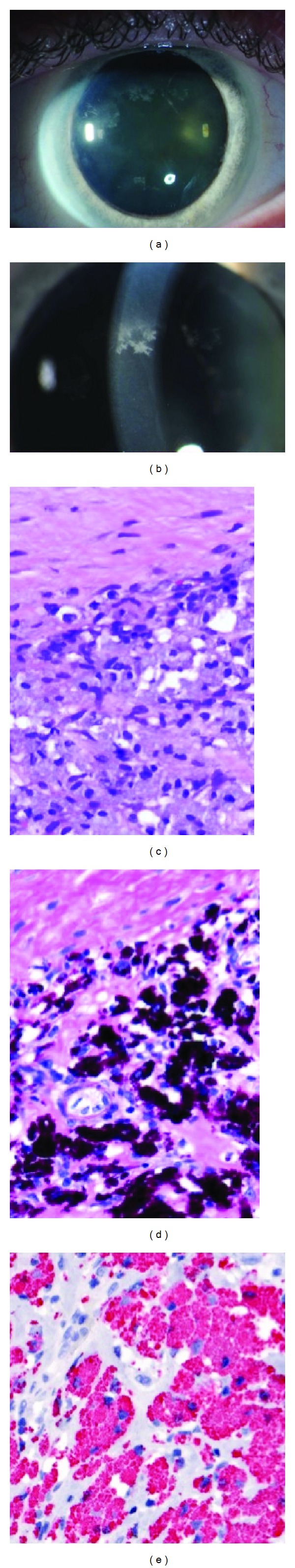
Three months after initial presentation, crystalline deposits are evident at the level of the corneal endothelium (a)-(b). Several months later, numerous foamy macrophages were identified on hematoxylin and eosin (H&E) stained aortic valve tissue (c) that stained robustly with periodic acid-Schiff diastase (d). The presence of organisms was confirmed by polyclonal anti-*T. whipplei* antibody (e). ((c)–(e)) courtesy of The Centers for Disease Control, Atlanta, GA.

**Figure 3:**
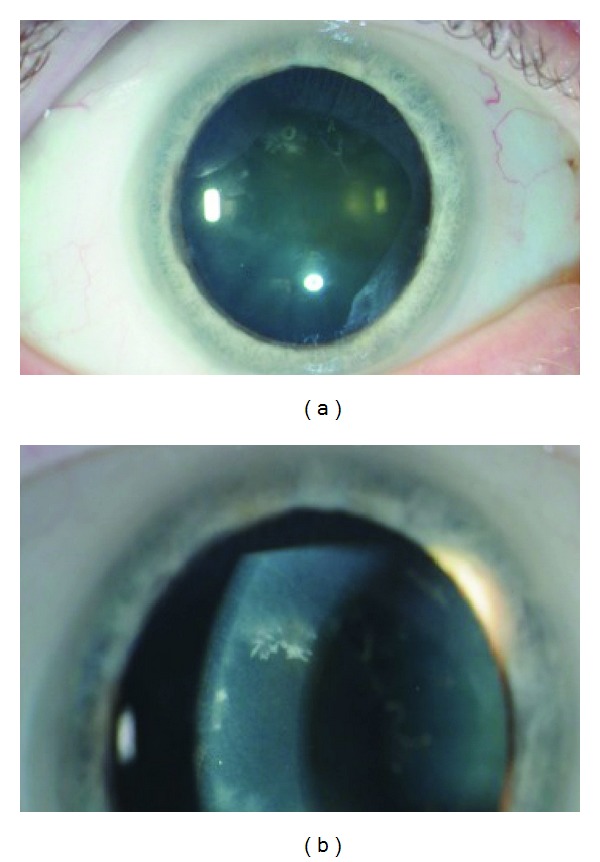
Photographs 17 months after initial presentation demonstrating fading of the endothelial crystalline deposits in the right eye (a-b).

Despite treatment with topical, oral, and periocular corticosteroids OD, inflammation persisted and whitish, fluffy endothelial deposits near the limbus in both eyes and an infiltrating “snowflake-like” crystalline keratopathy at the level of the endothelium OD were observed (Figure 2). Injection of intravitreal vancomycin and ceftazidime was performed given the suspicion of infection and aqueous fluid was cultured for bacteria, fungus, acid fast bacilli and PCR testing was performed for cytomegalovirus (CMV), Epstein-Barr virus (EBV) and VZV. In vitro response of aqueous fluid to mycobacterium tuberculosis was indeterminate and all remaining tests were negative. Further serological testing revealed negative antibodies for *Toxoplasma*, *Leptospira*, Hepatitis A, B, and C. EBV serology demonstrated evidence of past infection. Serum protein electrophoresis was unremarkable. A QuantiFERON gold test for *mycobacterium tuberculosis* was negative. A diagnostic and therapeutic vitrectomy was performed and testing of vitreous for *Borrelia burgdorferi* PCR and cytology was unrevealing. After cessation of corticosteroid treatment, the crystalline keratopathy (Figure 3) faded and intraocular inflammation resolved. Subsequent cataract surgery OD was performed without complication. 

Concurrent with his ocular inflammation, he was evaluated for decreased appetite, intermittent abdominal pain, and a 20-pound weight loss. Computed tomography (CT) of the abdomen and pelvis showed diffuse mild fatty infiltrates of the liver and borderline cardiomegaly. Lab work showed leukocytosis, which was felt to be reactive, iron deficiency anemia, and low folate levels. C-reactive protein (6.25) and ESR (34) were both elevated. He underwent genetic testing for hematological disorders and flow cytometry which were both unremarkable. He was started on iron and folate supplementation. He underwent upper GI endoscopy which showed a small sliding hiatal hernia and a colonoscopy which showed internal hemorrhoids. He underwent an upper GI biopsy demonstrated Barrett's esophagus. Thyroid testing and a cosyntropin stimulation test for adrenal insufficiency were normal.

Excisional biopsy was performed for an enlarged nontender right inguinal lymph node. Flow cytometry of the lymph node demonstrated a monoclonal population of lambda-restricted B cells with an immunophenotype consistent with chronic lymphocytic leukemia (CLL)/small lymphocytic lymphoma. However, hematoxylin and eosin (H&E) sections of the lymph node did not show effacement of architecture and immunohistochemical stains did not detect sheets of B cells, characteristics usually present in CLL. These findings were suspicious but not diagnostic for CLL/small lymphocytic lymphoma. Histopathology also demonstrated scattered and grouped macrophages with periodic acid-Schiff- (PAS-) positive inclusions suggestive of *Tropheryma whipplei* infection.

He developed worsening dyspnea due to decompensated valvular heart disease with biventricular heart failure. Echocardiography confirmed severe aortic regurgitation with mild left ventricular enlargement and an ejection fraction of 50%. Left heart catheterization showed no obstructive coronary disease. He underwent aortic valve replacement with a bi-leaflet mechanical valve. Intraoperatively, diffuse pericarditis was noted. 

He was started on high-dose trimethoprim-sulfamethoxazole for presumed Whipple's disease. A CT of the abdomen and pelvis was obtained which showed retroperitoneal pelvic inguinal adenopathy. The lymph node and aortic valve pathology blocks were sent to the Centers for Disease Control (CDC, Atlanta, Georgia) for review, and the presence of *T. whipplei* was confirmed by PCR, immunohistochemistry (immunoalkaline phosphatase technique), and PAS staining that showed PAS-positive diastase-resistant organisms within macrophages (Figure 2).

Despite initial improvement in appetite and weight gain, he gradually developed an aortic perivalvular leak and severe aortic insufficiency thought to be secondary to possible re-infection of the mechanical valve. He underwent another aortic valve replacement, ascending aortic replacement, and a 2-vessel coronary artery bypass. The prosthetic valve was sent to the CDC and also showed evidence of *T. whipplei* infection by PCR, PAS, and immunohistochemistry stains. He developed complete heart block and ultimately expired due to cardiogenic shock and cardiac arrest 2 years after his initial presentation for decreased vision. 

## 3. Discussion

The last review of ocular Whipple's disease was published more than a decade ago and despite recent advances in detection, delay in diagnosis remains common [2, 4]. Whipple's disease was first reported in 1907 and is a rare, chronic multiorgan bacterial infection caused by *T. whipplei* [1]. Males are more commonly affected (87%) [5], and mean age of onset is in the fifties [6]. While untreated Whipple's disease typically progresses to cachexia and death [3], treatment with corticosteroids or immunosuppressive medications may lead to rapid clinical deterioration and/or more treatment-resistant infection which underscores the importance of early and accurate diagnosis [2, 7]. 

Whipple's disease has a highly variable systemic presentation. The most common features include weight loss (93%), diarrhea (81%), and arthralgias/arthritis (73%) [5]. The joint involvement may be polyarticular, migratory, intermittent, and seronegative. Other gastrointestinal findings may also be present, including abdominal pain, occult bleeding, hepatosplenomegaly, and ascites. Neurologic involvement, which occurs in about a third of patients, portends a worse prognosis, as more than 25% die within four years [5]. Neurologic findings include cognitive changes, dementia, depression, and personality changes [5]. Neuro-ophthalmic findings occur commonly and include ophthalmoplegia, papilledema, gaze palsies, and nystagmus. Lymphadenopathy occurs in 52% of patients and usually involves the mesenteric lymph nodes, while peripheral lymphadenopathy is rare [5]. Fevers, pleural effusions, pulmonary infiltration, pericarditis, and culture negative endocarditis may also occur. 

Ocular involvement is uncommon, but increasingly recognized. In one report, ocular disease was present in 19 of 696 (2.7%) patients with confirmed systemic *T. whipplei* infection [8]. In 2001, Chan et al. reviewed 77 cases of ocular Whipple's disease [4]. They reviewed three groups: neuroophthalmic findings only (46 patients), neuro-ophthalmic and intraocular findings (10 patients), and intraocular findings only (21 patients). Since their review, nine additional cases of intraocular Whipple's disease have been reported [9–17]; thus, there are approximately forty reported cases of Whipple's disease with intraocular involvement to date. 

Ocular findings usually occur in the setting of prominent neurological, gastrointestinal, or other systemic symptoms, but some cases have been preceded by only mild arthralgias and/or nonspecific constitutional complaints [9, 10, 18, 19]. Ocular involvement is usually bilateral, but unilateral involvement has been reported [11]. Virtually any anatomic location of the eye can be involved, but vitritis is the most consistently reported finding [11, 18]. Anterior segment involvement may include keratitis, iritis, iris nodules, and peripheral corneal opacities and neovascularization [20, 21]. Posterior segment findings include pars plana snowbanks, snowballs, chorioretinitis, retinal vasculitis and capillary nonperfusion, cystoid macular edema, branch retinal artery occlusions, cotton wool spots, retinal and vitreous hemorrhages, optic nerve swelling, and choroidal folds [10, 13, 15, 22]. 

Ocular features of Whipple's disease overlap with many other conditions, but intraocular crystalline deposits have been described in approximately 13% (5 of 40) of reported cases to date (Table 1) [9, 14, 18, 20, 22]. Rickman et al. described a case of Whipple's disease with white, fluffy endothelial precipitates on the cornea [20]. Williams et al. described a case with crystalline deposits on the iris, intraocular lens, and capsular bag [18], and similar deposits have been reported in the anterior chamber [9], the retina [14], and subretinal or choroidal space [22]. To our knowledge, we are the first to describe crystalline keratopathy in Whipple's disease, but lack of previous association may have led to underreporting. Other established associations of crystalline keratopathy were either ruled out in this case or not considered directly causative and include infection (fungal, bacteria, and herpes simplex virus), Schnyder's crystalline dystrophy, tyrosinemia, gout, multiple myeloma, monoclonal gammopathy, and Waldenstrom's macroglobulinemia. Crystalline deposits in Whipple's disease are theorized to result from the high polysaccharide and peptidoglycan content in the *T. whipplei* bacillus [18]. Lack of awareness of this association, despite review of this case by several corneal experts, resulted in considerable delay in diagnosis.

There have been multiple associations between systemic *T. whipplei* infection and monoclonal B cell proliferations [23, 24]. It is unknown if this represents a reactive proliferation or Whipple's disease-related B-cell lymphoma [23], but many of these studies have not shown definitive evidence of lymphoma [23, 24]. The clonal population improved after treatment of the disease in some [24], but not others [23]. Our patient had a monoclonal B-cell proliferation with lambda chains determined by flow cytometry. As seen in other cases, definitive lymphoma could not be confirmed. Lymphoproliferative disorders have been associated with crystalline keratopathy, including Waldenstrom's macroglobulinemia (B-cell lymphoma), multiple myeloma, and monoclonal gammopathies [25–28]. The combination of chronic inflammation and monoclonal B-cell proliferation seen in Whipple's disease may predispose to the development of intraocular crystals. 

*T. whipplei* is ubiquitous in the environment [5], but its source and transmission have not been determined, though some have suggested fecal-oral transmission [2]. It is estimated that 2–4% of people in France have asymptomatic carriage of *T. whipplei *and that this number increases to 12–15% in sewer workers or toddlers, also supporting a fecal-oral transmission [29, 30]. Genetic factors, including HLA-DRB1*13 and DQB1*06, have been implicated [31]. Studies have shown impaired Th1 responses [32] and low serum levels of interleukin-12p40 and tumor necrosis factor-*α* [7, 33] among patients with Whipple's disease. It may also be a disease that results from macrophage dysfunction [34]. 

Blood tests may reveal elevated ESR, anemia, thrombocytosis or leukocytosis, and eosinophilia, but these findings are nonspecific [5]. Several different methods are available to diagnose Whipple's disease, including tissue biopsy with light and electron microscopy, PCR testing, and immunocytochemical staining with antibodies to *T. whipplei.* Light microscopic analysis shows PAS-positive inclusions and diastase-resistant bacilli within macrophages. However, PAS-positive inclusions are not completely specific for Whipple's disease. Noncaseating granulomas may also be present and electron microscopy may reveal the trilaminar wall of *T. whipplei*.

Detection has dramatically improved with the molecular amplification of the 16S ribosomal RNA of *T. whipplei *by PCR [35] and cell culture of the organism [36]. PCR can be detected from various body sources (including cardiac valves) [5]. It can be detected in saliva, but false positives occur (19–35% in healthy subjects) [37, 38]. Other studies have found much lower rates in the saliva (0.6%) and stool (1.5%) of healthy individuals [5]. Studies have shown that PCR may be positive in people with other gastrointestinal conditions in 5–12% of specimens [5]. It may also be present in other body fluids. Immunohistochemical stains using anti-*T. whipplei* antibody, as used in our current case, may confirm the diagnosis. It provides a greater specificity for the detection of the bacillus [39], and it can be detected from various bodily fluids and specimens, including heart valves [5]. Although the detection of *T. whipplei* infection can be difficult, our diagnosis is supported by multiple lines of evidence (histological, immunohistochemical, and PCR) and independent confirmation by the CDC. 

The above methods have been adapted to diagnose ocular Whipple's disease. PCR from aqueous and vitreous fluid have established the diagnosis in several cases [9, 11, 12, 14, 15]. Vitreous biopsies have demonstrated PAS-positive inclusions in macrophages [4, 10] and nonspecific chronic inflammation [4]. Immunocytochemistry of aqueous and vitreous samples has also been used successfully [10]. Although intraocular fluid was not tested for *T. whipplei* in this case (due to lack of awareness), its systemic presence was confirmed, and all other plausible causes of keratopathy were excluded. Ocular Whipple's disease is a known entity and histopathologic confirmation, while desirable, was neither possible in this case due to the family's decline of an autopsy nor necessary given the certainty of cause of death. Nevertheless, the unusual ocular presentation of this case in concert with systemic findings supports the diagnosis.

Several antibiotics have been used for treatment. Tetracycline and doxycycline have been used successfully, though they may be associated with a higher relapse rate (28%) [5]. Many consider trimethoprim-sulfamethoxazole to be the first-line therapy, with continued treatment for a total of 12 to 18 months. Relapse may occur, however, even after effective treatment with antibiotics [40] in 2% to 33% of cases [5]. During relapses, PCR testing for *T. whipplei* tends to be negative [40]. Neurologic recurrences confer a poor prognosis [41] and previous immunosuppressive treatment is significantly associated with recurrences [40]. 

In summary, the diagnosis of Whipple's disease remains challenging and a high index of suspicion is required. Delay in diagnosis can result in mortality, and administration of corticosteroids or other immunosuppressive medications may result in rapid deterioration and/or more treatment resistant disease. Ocular signs and symptoms are usually nonspecific, but the appearance of intraocular crystals or crystalline-like deposits may be a unique feature of this disease and may allow earlier diagnosis and more timely intervention of future cases.

## Figures and Tables

**Table 1:** Review of cases reporting intraocular crystalline or crystalline-like deposits in the setting of Whipple's disease.

Report	Age	Gender	Location of deposits	Diagnosis	Systemic findings at presentation
Current case	49	Male	Corneal endothelium	Heart valve and lymph node Bx	Arthralgias
Thaler et al. [9]	56	Male	Anterior chamber	Vitreous and duodenal PCR, duodenal Bx	Arthralgias
Williams et al. [18]	62	Male	Intraocular lens, capsular bag, iris	Vitreous and lens capsule biopsy and PCR	Arthritis Fevers
Lim et al. [14]	23	Male	Preretinal or inner retinal	Vitreous PCR, jejunal Bx	Anemia, lymphadenopathy, diarrhea, malnutrition
Rickman et al. [20]	59	Female	Corneal endothelium	Vitreous biopsy, EM, PCR	Arthritis
Avila et al. [22]	33	Female	Subretinal	Small bowel biopsy showing PAS positive granules, EM	Fever, anorexia, diarrhea, weight loss

Bx: biopsy; PCR: polymerase chain reaction; EM: electron microscopy; PAS: periodic acid-Schiff.
